# Quantum-Optically Enhanced STORM (QUEST) for Multi-Emitter Localization

**DOI:** 10.1038/s41598-018-26271-1

**Published:** 2018-05-18

**Authors:** Marc Aßmann

**Affiliations:** Experimentelle Physik 2, Technische Universität Dortmund, 44227 Dortmund, Germany

## Abstract

Super-resolution imaging has introduced new capabilities to investigate processes at the nanometer scale by optical means. However, most super-resolution techniques require either sparse excitation of few emitters or analysis of high-order cumulants in order to identify several emitters in close vicinity. Here, we present an approach that draws upon methods from quantum optics to perform localization super-resolution imaging of densely packed emitters and determine their number automatically: Quantum-optically enhanced STORM (QUEST). By exploiting normalized photon correlations, we predict a localization precision below 30 nm or better even for closely spaced emitter up to a density of 125 emitters per *μ*m at photon emission rates of 10^5^ photons per second and emitter. Our technique does not require complex experimental arrangements and relies solely on spatially resolved time streams of photons and subsequent data analysis.

## Introduction

The development of super-resolution microscopy resulted in a drastic improvement of optical imaging capabilities by breaking the diffraction limit which restricts the resolution of traditional wide-field or confocal microscopy techniques to approximately half of the emitted wavelength. Besides technologically challenging techniques like stimulated emission depletion microscopy (STED)^1,2^ and structured illumination microscopy (SIM)^3^, stochastic localization techniques such as PALM^4^, FPALM^5^ or STORM^6^ have found widespread usage. However, they suffer from long acquisition times due to the requirement that only few fluorophores may emit light at any instant because simultaneous overlapping emission prevents correct localization of the individual emitters. This limitation has been overcome by fluctuation-based techniques such as independent component analysis^7^ and techniques based on dedicated multi-emitter fitting procedures^8,9^, which require a good initial estimate of the number of emitters involved and other parameters, or SOFI^10^, which relies on the calculation of temporal cumulants or spatio-temporal cross-cumulants. For the latter technique, using the *n*th-order cumulant results in an effective point spread function (PSF) scaling as the *n*-th power of the original PSF, results in a significant $\sqrt{n}$ improvement of the resolution, but shows non-trivial brightness scaling with *n*, which tends to mask dim emitters and becomes computationally expensive for large *n*. It is also not a localization technique. Therefore, it would be desirable to have a localization technique that is sensitive to fluctuations, but not directly sensitive to the emitted intensity. This question has been addressed before in different context. In fact, the idea of being able to characterize the nature of a light field in terms of coherence and independent of its intensity, wavelength components or polarization was a basic idea driving the field of quantum optics and the theory of optical coherence^11^. Here, we propose an alternative approach towards localization super-resolution imaging of emitters with spatially overlapping emission spectra based on an quantum-optical approach that relies on normalized correlation functions. Our technique is tailored for the next generation of super-resolution detectors based on single photon avalanche diode arrays^12^ or single photon fiber bundle cameras^13^ instead of CCD cameras. This new kind of detector allows for a modular approach towards super-resolution imaging that makes it possible to use different algorithms for different parts of the image or different times. The technique demonstrated here is tailored to be such a module for small regions, where presumably a large number of overlapping emitters is located and the emitted photon number is locally large. It can still be used as a standalone module to perform imaging under standard conditions, but for imaging spatially extended regions conventional high density localization techniques can achieve similar precision already at signal levels that are smaller by two orders of magnitude. Still, as a proof of principle, to highlight the complementarity to techniques like SOFI and to demonstrate the mode of operation of QUEST without having to add a different technique as a basis module, it will be used as a standalone module in the following. However, the reader should be reminded that this is not its designated mode of operation. The next section describes the theory underlying our approach.

## Results

### Theory

**Figure 1:**
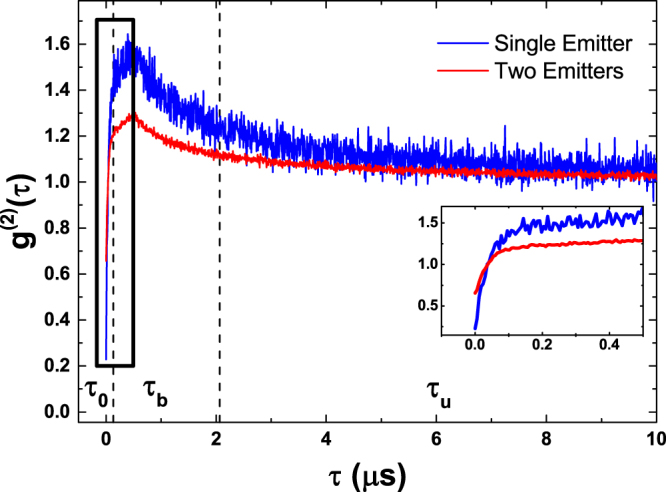
Normalized second-order intensity correlation traces for a single emitter (blue line) and a superposition of two distinguishable emitters (red line). For the simulations, a power law blinking model with exponents of *α*_*on*_ = 1.7 and *α*_*off*_ = 1.78 have been used. Details on the chosen model can be found in the methods section. Vertical dashed lines mark the borders between delay ranges where antibunching (*τ*_0_, *g*^(2)^(*τ*_0_) < 1), blinking (*τ*_*b*_, *g*^(2)^(*τ*_*b*_) > 1) and uncorrelated emission (*τ*_*u*_, *g*^(2)^(*τ*_*u*_) ≈ 1) become predominant. The inset shows a close-up view of the region marked by the black rectangle.

In quantum optics, the conditional probability to detect a second photon at a delay *τ* after a first photon has been detected is given by the normalized second-order correlation function of the total light field:

1 $${g}^{\mathrm{(2)}}(\tau )=\frac{\langle :n(t)n(t+\tau ):\,\rangle }{\langle n(t)\rangle \langle n(t+\tau )\rangle },$$

where the double stops denote normal ordering of the bosonic field operators underlying the photon numbers. This normal ordering ensures that the destructive nature of the photon detection process is respected: A single photon cannot be detected twice. *g*^(2)^ (*τ*) can be interpreted as the count rate of photon pairs at a delay *τ* divided by the product of the mean photon count rates at the corresponding times. The latter equals the photon pair count rate expected if photons were emitted completely independent of each other. This allows one to distinguish three basic cases. For *g*^(2)^ = 1, photons are indeed emitted independent of each other. For *g*^(2)^ < 1 the detection of the first photon results in a reduced probability to detect a second photon. This antibunching is typical for single photon emitters, where the emission of a photon blocks this emitter until it has returned to the excited state and can emit a photon again. *g*^(2)^ > 1 corresponds to strong fluctuations of the photon number. At times where the emitted intensity is larger than the mean photon number, the photon pair detection rate increases superlinearly with the photon number and thus results in statistically correlated photon emission. This behavior is typical for blinking emitters^14,15^, which switch back and forth between a dark and a bright state. Many fluorophores used in microscopy such as single molecules or colloidal quantum dots are single photon emitters^16–18^ and their emission shows all of these effects. At short timescales *τ*_0_, the emission of a second photon is suppressed, at intermediate timescales of the order of the typical scale of blinking *τ*_*b*_, correlated emission occurs, while the emission for very long delays *τ*_*u*_ and for photons emitted from different emitters consists of statistically independent photons. A typical *g*^(2)^ (*τ*) trace of a single emitter acquired at a certain spatial position is shown in Fig. 1.

The antibunching at short delays *τ*_0_, blinking at intermediate delays *τ*_*b*_ and independent emission at large delays *τ*_*u*_ can be seen clearly. For comparison, *g*^(2)^ (*τ*) for a light field to which two emitters contribute equivalently is also shown. As can be seen, the antibunching and blinking effects are reduced in magnitude. Only photon pairs from the same emitter are correlated. Instead, those coming from different emitters are usually uncorrelated, which pushes the values of *g*^(2)^ (*τ*) closer to 1. In contrast to the emitted intensity or the unnormalized correlation function, *g*^(2)^ (*τ*) is thus directly sensitive to the overlap of different emitters. It should be noted that in order to determine the distribution of time delays of photon pairs, all photons detected at a certain pixel are taken into account and considered as pairs, not just consecutive ones.

QUEST is based on recording a spatially resolved map of *g*^(2)^ (*τ*, *x*, *y*) which yields detailed information about the spatial position of the emitters. One can then apply appropriate fitting of the second-order correlation function to recover the position of the emitters with high resolution. In order to apply QUEST, the following conditions must be met:

1. The fluorophore should either be a single photon emitter or show fluorescence blinking or both.
2. The pixels of the detector used should be smaller than the spatial extent of the point spread function of the imaging system used.
3. The fluorophores are independent emitters.
4. The detector used should be fast enough to resolve the antibunching timescale or see several on/off-cycles of the fluorophore during a frame.

**Figure 2:**
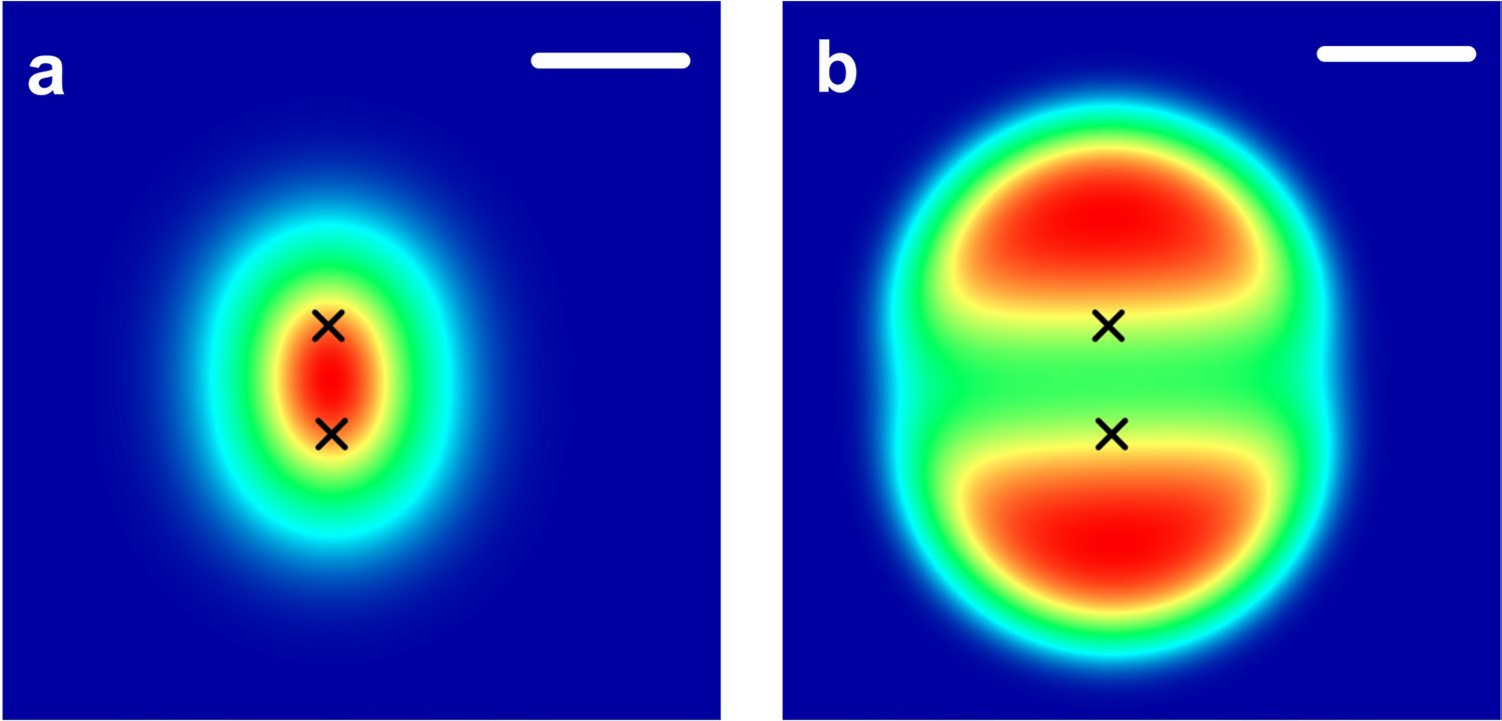
High-resolution correlation imaging of the emission of two emitters placed at a distance of 150 nm. Black crosses mark the position of the emitters. (**a**) Emitter fluorescence. (**b**) *g*^(2)^(*τ*_*b*_, *x*, *y*) blinking image. The antibunching image *g*^(2)^(*τ*_0_, *x*, *y*) looks exactly the same, but the deviations from the value of one have the opposite sign. (Scale bars: 200 nm).

In principle, both the antibunching signature *g*^(2)^ (*τ*_0_, *x*, *y*) and the blinking signature *g*^(2)^ (*τ*_0_, *x*, *y*) contain the full information about the spatial distribution of the emitters. In the following we restrict ourselves to a discussion of the blinking signature, as it usually shows larger photon count rates. Still, in experiments it will be most sensible to evaluate both quantities simultaneously. The combined information from both independent localization signatures will result in reduced localization error. In Fig. 2 we compare a standard optical fluorescence image to a *g*^(2)^ (*τ*_*b*_, *x*, *y*) blinking image for two emitters placed at a distance of 150 nm and assuming a point spread function that is Gaussian with a standard deviation of σ_PSF_ = 100 nm, which corresponds to one Airy unit of about 290 nm. The positions of the emitters are marked by black crosses. The left panel shows the emitted intensity. The emission from both emitters overlaps strongly and their individual position cannot be identified directly. The right panel shows the *g*^(2)^ (*τ*_*b*_, *x*, *y*) blinking image. A strong directional dependence is apparent. In the middle between the emitters, the value of *g*^(2)^ (*τ*_*b*_, *x*, *y*) is reduced significantly, while it increases in the region beyond the emitters.

This peculiar shape can be explained as follows: *g*^(2)^ (*τ*_*b*_, *x*, *y*) quantifies the normalized fluctuations of the intensity of the light and is independent of the mean intensity of the light field. For an individual emitter in the absence of any external noise, *g*^(2)^ (*τ*_*b*_, *x*, *y*) will thus take the same constant value for any position in space. In the presence of background noise or a second emitter, the distribution will change. *g*^(2)^ describes a conditional probability distribution for photon pairs. For *N* emitters, it will now consist of the individual second-order autocorrelations ${g}_{i}^{\mathrm{(2)}}$ of the emitters and the second-order crosscorrelations ${g}_{ij}^{\mathrm{(2)}}$ between the emitters weighted by the relative probability that these two emitters contribute to a photon pair. Based on these assumptions a model fit function for *g*^(2)^ (*τ*, *x*, *y*) can be found. We assume *N* contributions to the fluorescence signal, where the signal for the first *N* − 1 emitters at each position (*x*, *y*) is given in terms of their brightness *I*_*i*_ and location (*x*_*i*_, *y*_*i*_) and a Gaussian point spread function:

2 $${I}_{i}(x,\,y)={I}_{\mathrm{0,}i}\exp (-\,\frac{{(x-{x}_{i})}^{2}+{(y-{y}_{i})}^{2}}{2{\sigma }_{i}})$$

for i ≠ N and

3 $${I}_{N}(x,\,y)={I}_{\mathrm{0,}N}$$

describes the background noise, which we treat as an emitter with a flat point spread function. The spatially resolved total fluorescence signal intensity amounts to:

4 $${I}_{tot}(x,\,y)=\sum _{i\mathrm{=1}}^{N}{I}_{i}(x,\,y).$$

The relative contribution of a single emitter to the total emission at any position is given by:

5 $${r}_{i}(x,\,y)=\frac{{I}_{i}(x,\,y)}{{I}_{tot}(x,\,y)}.$$

The total second-order correlation function consists of the second-order autocorrelations ${g}_{i}^{\mathrm{(2)}}$ of the individual emitters and the second-order crosscorrelations ${g}_{ij}^{\mathrm{(2)}}$ between the emitters, both weighted by the ratios *r*_*i*_:

6 $${g}^{\mathrm{(2)}}(x,\,y)=\sum _{i=1}^{N}{g}_{i}^{\mathrm{(2)}}{r}_{i}^{2}(x,\,y)+\sum _{i,j=\mathrm{1,}\,j\ne i}^{N}{g}_{ij}^{\mathrm{(2)}}{r}_{i}(x,\,y){r}_{j}(x,\,y).$$

This is the fitting function. Here, we assume the background noise to be uncorrelated, so ${g}_{N}^{\mathrm{(2)}}=1$, and all emitters to be independent, so ${g}_{ij}^{\mathrm{(2)}}=1$. Within this work, we assume all emitters besides the noise to have the same brightness *I*_0_ and the same value of ${g}_{i}^{\mathrm{(2)}}={g}_{0}^{\mathrm{(2)}}$, but this assumption can be dropped for real systems. It should be noted that the fit formula is valid both for *τ*_0_ and *τ*_*b*_.

Knowledge of the fitting function for *g*^(2)^(*τ*_*b*_, *x*, *y*) is a necessary prerequisite for successful position reconstruction, but also a good estimate of the total number of emitters present within a certain area of interest will simplify reconstruction significantly. QUEST is able to automatically determine this number by first evaluating the total antibunching signature *g*^(2)^(*τ*_0_) integrated over all pixels within a region of interest. It is well known that the equal-time second-order correlation function ${g}^{\mathrm{(2)}}\mathrm{(0)}=1-\,\frac{1}{N}$ for *N*-photon states^19^. This property of the light field is routinely used to identify single photon sources^20^. In very good approximation, the same value of *g*^(2)^(0) holds true for *N* single-photon emitters even if blinking is present^21^. Therefore, the value of the equal-time correlation function at zero delay may be used to estimate the number of emitters present, provided that the temporal resolution of the detector is sufficient to resolve the correct behavior at short delays. It should be noted that number estimation becomes unreliable for large numbers of fluorophores as the values *g*^(2)^(0) become close to one. Therefore, for wide field imaging, QUEST can be performed using iterative segmented spatial mapping of *g*^(2)^(0) such that the QUEST algorithm estimates the number of emitters inside a region of interest and if it is too large to yield reliable results automatically subdivides it into two smaller regions of interest containing fewer emitters. Repeating this procedure until each segment contains a small number of emitters and consecutive reconstruction of the emitter locations in each individual segment will then provide the full list of emitter positions. It should be noted that this segmented approach can be used to automatically investigate several region which contain the same emitters, but are slightly shifted with respect to each other. This technique allows for more robust emitter localization for emitters of varying brightness.

#### Results of simulations

**Figure 3:**
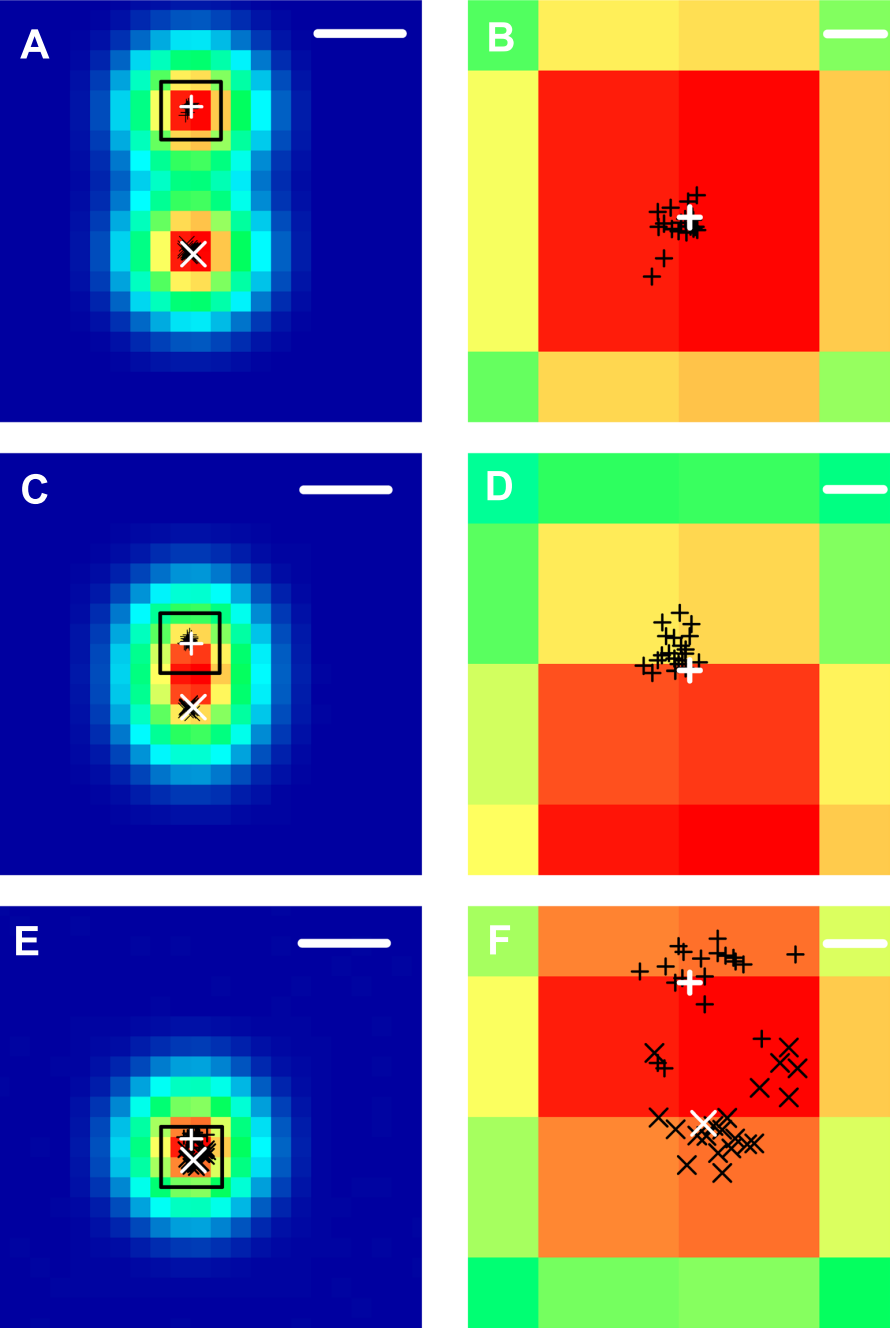
Reconstruction of two emitter positions at varying distances using QUEST. (**A**,**C**) and (**E**) show the total emitted fluorescence of two emitters placed at distances of 350, 150, and 50 nm, respectively. White crosses mark the real positions of the emitters. Black crosses show the positions of the emitters reconstructed using QUEST for 20 different imaging cycles (scale bars: 200 nm). (**B**,**D**) and (**F**) show magnified views of the boxed regions in the left panel (scale bars: 20 nm).

We first test the QUEST localization technique on simulated data for two emitters placed at varying distances. For the simulation, we again assume a Gaussian point spread function with σ_PSF_ = 100 nm corresponding to one Airy unit of about 290 nm and simulate a time-tagged stream of photons over a duration of 15 seconds. In order to show the robustness of QUEST to noise, we choose a large dark count rate of about 200000 counts per second that is comparable to the photon emission rate per emitter. We investigate distances both larger and shorter than the diffraction limit. Figure 3 shows the results of simulations for emitters placed at distances of about 350 nm, 150 nm and 50 nm apart. The color plots show the total emitted fluorescence as acquired in typical experimental approaches such as total-internal-reflection fluorescence microscopy. White crosses mark the real positions of the emitters and black crosses show the reconstructed positions using QUEST for 20 imaging cycles. Magnified views of the boxed sections are shown in the right panel.

For the larger distances, the mean position localization error per emitter as defined by the mean absolute distance between the emitter position used in the simulations and the reconstructed emitter position amounts to about 10 nm with a standard deviation of 5.5 nm per dimension. For the smallest distance, we find a slightly larger mean absolute position localization error of 20 nm per emitter with a standard deviation of 14.5 nm. It is worth noting that for small emitter distances, the reconstructed fluorophore positions are not distributed symmetrically around the real position, but show a bias towards the outward direction. This effect is clearly demonstrated in Fig. 3D and is a consequence of the strongly asymmetric shape of the fitting function. As the normalized second-order correlation function depends non-linearly on the distance between the emitters, shifting two emitters closer together changes the resulting *g*^(2)^(*τ*, *x*, *y*) more strongly than shifting them further apart by the same amount. This effect can in principle be avoided by specialized fitting procedures or maximum likelihood approaches.

**Figure 4:**
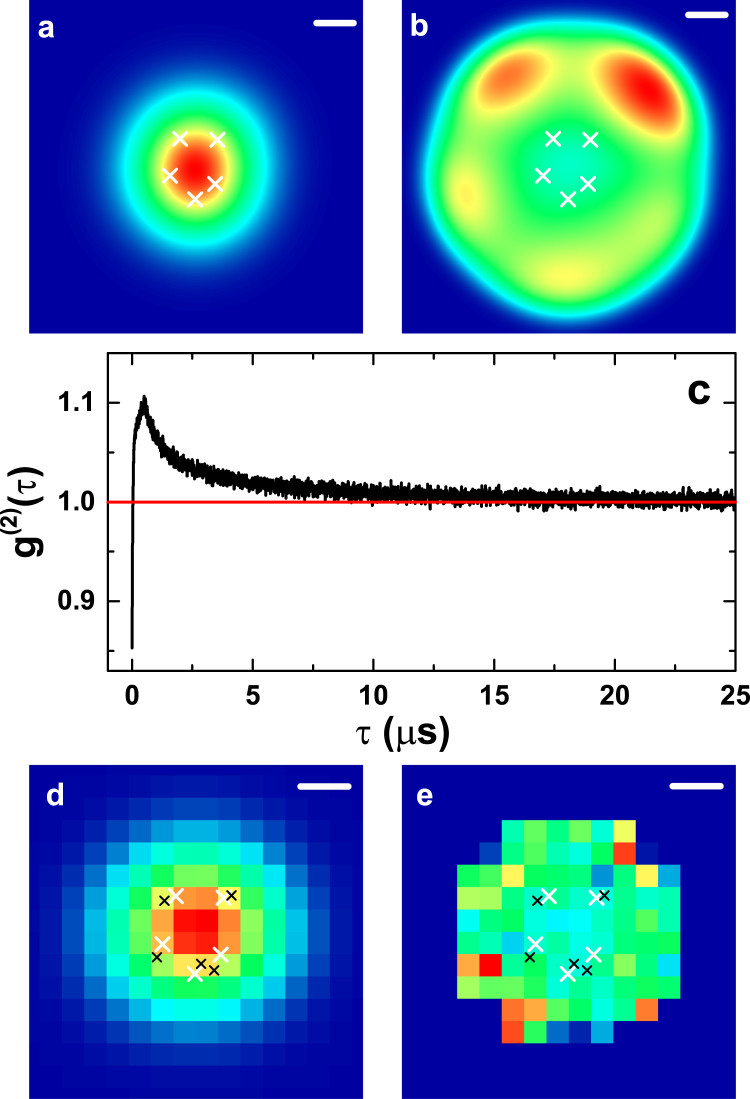
Reconstruction of multiple emitters demonstrated for five emitters placed in a small area of 2*σ*_PSF_ × 2*σ*_PSF_. (**a**) and (**b**) show theoretical high-resolution images of total fluorescence and *g*^(2)^(*τ*_*b*_). White crosses denote the real positions of the fluorophores. (**c**,**d**) and (**e**) show simulation results and corresponding position reconstruction. (**c**) represents the spatially integrated *g*^(2)^(*τ*) that allows us to determine the number of emitters present. (**d**) and (**e**) show the simulated intensity and *g*^(2)^(*τ*_*b*_). Black crosses represent the reconstructed fluorophore positions. Scale bars: 100 nm.

In order to evaluate the validity of our approach for densely placed emitters with strongly overlapping point spread functions and present the detailed operating principle of QUEST, we have also simulated 5 emitters placed within a 2σ_PSF_ × 2σ_PSF_ square region, which would correspond to a local emitter density of *ρ* = 125 emitters *μ*m^−2^ in the central region. The total emitted fluorescence is shown in Fig. 4a. Only a single large spot can be seen, from which the positions of individual emitters cannot be identified reliably. In contrast, the *g*^(2)^(*τ*_*b*_, *x*, *y*) map displayed in Fig. 4b shows a more complicated behavior. In the central region, where the emission of all the emitters overlaps, *g*^(2)^(*τ*_*b*_, *x*, *y*) shows small values, but at several positions away from the peak of the intensity distribution large values of *g*^(2)^(*τ*_*b*_, *x*, *y*) can be found. In analogy to the case of two emitters shown in Fig. 2, at these positions, one emitter provides the main contribution to the detected intensity. The direction and magnitude of this increase in *g*^(2)^(*τ*_*b*_, *x*, *y*) are good indicators of the emitter positions.

**Figure 5:**
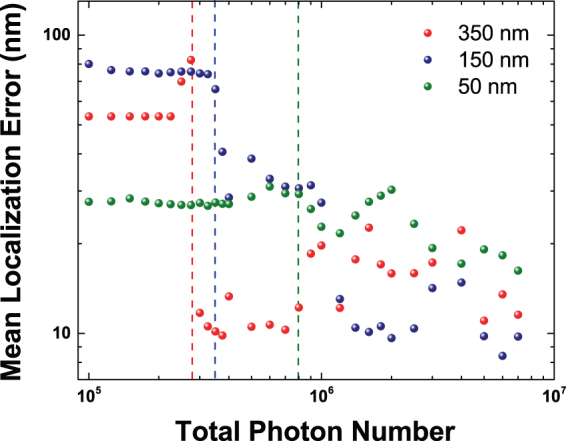
Mean localization error in dependence on the number of photons used as an input for QUEST for two fluorophores placed at varying distances. A clear threshold for efficient reconstruction can be identified for every distance. Its onset is marked by dashed lines.

We now perform a simulation for this geometry based on individual emission events for a detector with an effective resolution of 50 nm per pixel and apply QUEST in order to reconstruct the fluorophore positions. Simulation results are shown in Fig. 4c–e. Figure 4c shows the spatially integrated *g*^(2)^(*τ*), which we use to determine the number of emitters present in the image. The antibunching value of *g*^(2)^(*τ* = 0) amounts to 0.853. Taking the dark count rate of 14.5% into account results in a corrected *g*^(2)^(*τ* = 0) of 0.798, which directly translates into an emitter number estimate of 4.95 and correctly indicates the real number of five emitters. We now use the *g*^(2)^(*τ*, *x*, *y*) map shown in Fig. 4e as input for QUEST and reconstruct the fluorophore positions indicated by the black crosses. The positions are determined well with a mean localization error of 26.9 nm. As can be seen in Fig. 4e, we have included a significance threshold in determining the map of *g*^(2)^(*τ*_*b*_). We disregard photon pairs with a delay larger than 50 *μ*s and consider pixels with a photon pair count rate of less than 100 Hz as strongly dominated by background noise which do not yield a reasonable estimate of the second-order correlation function at that position. These points have been set to a value of 1 in the map and are not considered during the fitting procedure. Accordingly, it is interesting to investigate whether it is beneficial to have longer integration times and include regions with low photon count rates while applying QUEST. To this end, we again investigate the mean localization error for the two fluorophores at varying distances already shown in Fig. 3 for different total photon numbers per localization image. Each of the emitters and the background noise contribute approximately the same number of detection events to the photon number. Results are shown in Fig. 5. All curves show a clear onset of efficient position reconstruction marked by a significant decrease of the mean localization error towards values on the order of 10 to 20 nm in the range between photon numbers of 300,000 and one million. The onset shifts to smaller photon numbers for larger distances. Increasing the photon number to even larger values does not improve the reconstruction significantly. The results may even become worse. This behavior may seem surprising, but can be explained by the fact that additional pixels reach the threshold value of 100 Hz photons for larger count rates. The errors of the values of *g*^(2)^(*τ*_*b*_) at these pixels may still be moderately large, which results in reduced localization accuracy. Dedicated statistical approaches may improve the treatment of these pixels, but are out of the scope of the present manuscript.

**Figure 6:**
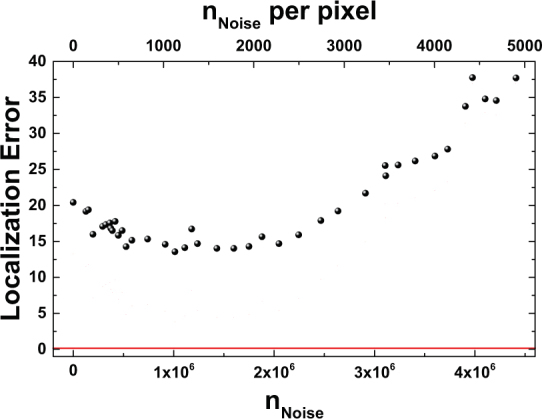
Mean localization error per emitter for 10^6^ total signal photons originating from two emitters at a distance of 350 nm superposed with varying numbers of randomly distributed noise photon numbers *n*_*Noise*_. With the exception of 0 noise photons, all data points are averaged over 30 realizations of different noise distributions. The peak signal intensity at a single pixel amounts to 19242 counts. For comparison the red line shows the ideal single emitter shot-noise limit that can be achieved using STORM in the absence of background noise.

Another factor to consider is the influence of the background noise level on localization accuracy. An exact treatment is way beyond the scope of this manuscript, but the general effect can be explained well qualitatively. As an example, Fig. 6 shows the localization error per emitter for two emitters placed 350 nm apart in the presence of noise. For all data points, the same basic simulation of one million signal photons has been used and superposed with different noise datasets with a total number *n*_*Noise*_ of noise counts and the same total duration as the signal datasets. 30 realizations of noise have been used for each data point. It is striking that in contrast to most other approaches to super-resolution imaging, the localization precision initially increases in the presence of noise roughly up to the point where the noise count rate equals $\sqrt{2}$ times the signal count rate and deteriorates when increasing the noise count rate even further. In order to understand this kind of effect, it is important to keep in mind that QUEST does not map the absolute emitted intensity, but the overlap of the emission of different emitters and their relative intensities at different points. In the simplest case, background noise can be considered as an additional homogeneous emitter. Considering for example a single isolated emitter, one immediately sees that background noise may even enhance localization accuracy. The normalized second-order correlation function for a single emitter is uniform all over space and thus yields no information about the emitter position at all. Adding some constant background noise will result in spatially varying relative contributions of signal and noise and thus result in a second-order correlation function that shows a peak at the emitter position and enhanced localization precision. Similar considerations apply for larger numbers of emitters.

**Figure 7:**
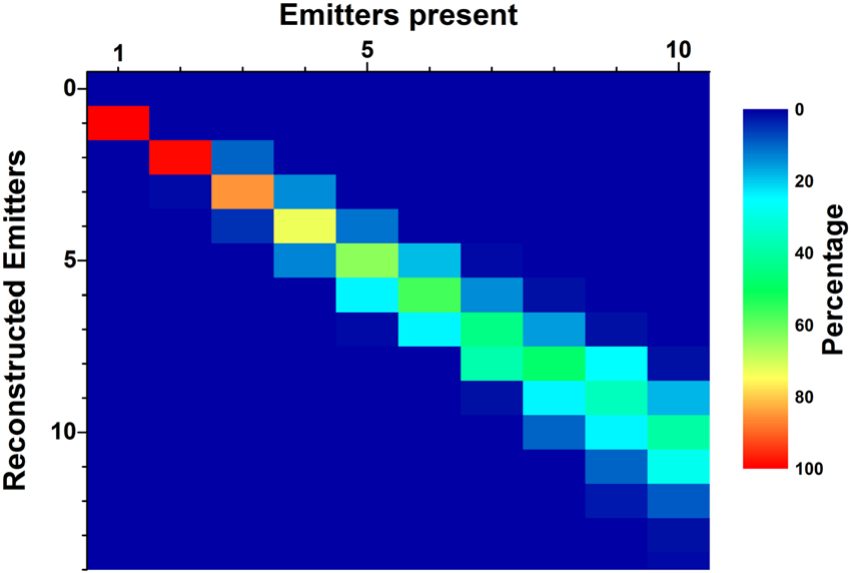
Performance of emitter number recognition for emitters placed randomly in an area of 1 *μ*m^2^. Each column gives the relative frequency distribution for a fixed emitter number. For the simulations, an average photon number of 6000 photons per emitter has been assumed.

Finally, it is instructive to discuss the performance of emittter number estimation based on the normalized second-order correlation function. We performed simulations using a varying number of emitters placed randomly inside an area of 1 *μ*m^2^ and considered an average photon number of 6000 per emitter. 100 simulations were performed for each fixed emitter number. The columns in Fig. 7 show the distribution of emitter number estimates for each fixed emitter number. Recognition is close to optimal for small emitter numbers, but even up to 10 emitters, the emitter number estimate distribution is moderately narrow and still centered on the real number of emitters. However, there is a slight trend towards overestimated emitter numbers for many emitters, which can be traced back to the range of values of the second-order correlation function that corresponds to a certain emitter number becoming smaller when placing more emitters. However, this problem can in principle be overcome by using tailored sectioning approaches.

## Discussion

We have proposed a quantum optics based super-resolution technique suitable for localization of multiple simultaneously emitting fluorophores with overlapping point spread functions that still works for local emitter densities on the order of 100 emitters *μ*m^−2^ and is strongly sensitive to the relative orientation of the emitters. Our approach is tailored for having low experimental requirements, is robust with respect to the experimental data acquisition process and is computationally efficient as it does not require an underlying grid. The experimental procedure amounts to recording a spatially resolved time-stream of photons under standard optical excitation conditions and the choice of fluorophores and optics used is not critical as long as the fluorophores are bright enough. The critical difference to standard STORM setups lies in replacing the CCD detector with a single photon fiber bundle camera, which requires no further changes to the experimental setup. The method identifies the number of emitters present and does not require a priori knowledge of this number. The precision and speed of the method, however, strongly depend on the specific implementation of the recovery algorithm used and we want to emphasize that we only present the basic technique here which leaves plenty of opportunities for improvement in terms of data analysis. First, the least-squares fitting approach here is easy to implement, but for real detectors with possibly asymmetric point spread functions a maximum-likelihood approach^9^ also taking the total intensity distribution into account might be more suitable. We expect that a more specialized reconstruction approach may result in improved localization precision. For example, as can be seen in Fig. 3 the strongly asymmetric shape of the *g*^(2)^(*τ*_*b*_, *x*, *y*)-map for two emitters with overlapping point spread function results in a position estimate that is not centered on the emitters, but systematically shifted away from the region of the overlap. This deviation may be corrected by tailored algorithms using weighted data. Further optimization might also be achieved by utilizing deep learning^22^ to find optimal reconstruction parameters or by using compressive sensing^23^ or other sparsity based algorithms with^24^ or without^25^ using an underlying grid. Also the photon count threshold used is a degree of freedom that may be optimized. For example approaches using weights depending on the photon count rates per pixel or using spatial regions of variable sizes for analysis are likely to outperform the basic least square fitting approach described here. It should also be noted that the completely symmetric Gaussian point spread functions used in the simulations are the worst possible case for emitter localization. Any asymmetry in the point spread function will make it easier to reconstruct the position of the fluorophores. Along the same lines, although we have only discussed 2D-imaging, intentionally added astigmatism could be used to render our method capable of 3D imaging in analogy to 3D STORM^26^, which should in principle allow for a depth resolution of at least 50–60 nm.

QUEST has the potential to perform super-resolution imaging at high frame rates up to real time imaging. The required integration times for QUEST will depend on the brightness and density of the fluorophore used. Acquisition times of few seconds seem reasonable for typical experimental parameters and sub-second data acquisition may become feasible for low background noise. However, due to the complex data analysis involved, achieving such fast acquisition rates will require hardware based online data analysis e.g. by applying field programmable gate arrays for fast real-time on chip coincidence counting and analysis^27^ such as on-chip fitting of the *g*^(2)^(*τ*)-curve for every pixel. Building upon such an architecture, further extensions might be considered. For example higher-order correlations are supposed to be even more sensitive to the emitter positions and taking cross-correlations between different pixels into account in a similar manner to SOFI^10^ may result in reduced noise and acquisition times. It should also be noted that QUEST is not necessarily limited to biological imaging and organic dyes. In some respects, quantum dots may be a better choice as the fluorescent emitter as tailoring their parameters allows some limited control over the blinking behavior and can reduce the disadvantageous low-frequency blinking^28^. Further, in order to utilize the fast frame rates available with ultrafast imagers, using emitters with matching photon emission rates would be advantageous. Therefore, QUEST lends itself rather to imaging in fields such as semiconductor physics, where quantum dots may be used rather than biological imaging. Along the same lines, one drawback of QUEST is that its usefulness depends strongly on the detector architecture employed and for standard detectors such as CCD cameras, it will not be overly useful as it does not perform well compared to established techniques in the limit of very small photon count rates.

It is instructive to compare our approach to other super-resolution techniques. It provides a tradoff between localization techniques such as STORM and super-resolution techniques based on unnormalized emitter noise properties. Regardless of whether the latter techniques rely on antibunching^29,30^ or blinking such as SOFI, they are usually not localization techniques like STORM, but may be cautiously called imaging techniques because they provide wide field images of quantities such as cumulants, which allow for better resolution than fluorescence images. Both kinds of techniques are known to have advantages and disadvantages^31^. Localization techniques typically yield higher resolution, but also have more strict requirements. The point spread function of the imaging system must be known in detail and simultaneous emission from overlapping emitters must be avoided, which renders data acquisition slow for large emitter densities. SOFI, on the other hand, has less strict requirements on knowledge of the imaging system and emitter properties. Further, simultaneous emission from densely packed emitters is not a problem for SOFI, so it has the potential for substantially faster data acquisition rates compared to STORM and for straightforward implementations of 3D imaging. However, as SOFI is based on higher-order cumulants, the intensity scale in SOFI images is necessarily strongly non-linear which may result in emitters with low intensity not being recognized. QUEST takes a position in the middle between these techniques. As the most important difference to SOFI, instead of cumulant images, QUEST records spatially resolved images of normalized second-order or higher-order correlation functions. Normalized correlation functions avoid the problem of emitters of weak intensity as is known from similar correlation-based super-resolution techniques that rely on confocal scanning^32^. Besides the effects of noise, every isolated emitter with the same emission profile will form exactly the same QUEST image. The normalization ensures that weak emitters also leave a significant trace for densely packed emitters that will show up as an anisotropy in the QUEST image. However, this advantage comes at the drawback that the QUEST image does not directly provide information about emitter positions. The positions of emitters do not correspond directly to maxima or minima in the QUEST image and must be recovered via fitting procedures which require information about the imaging system. However, due to the usage of normalized correlation functions, it requires little information about emitter properties such as emitter number or brightness. As a first approach, one may use iterative sectioning and include these quantities as fitting parameters, more sophisticated and precise strategies for emitter number estimation exist^33,34^ and may be optimized for QUEST images. QUEST is therefore similar to localization techniques, but has the additional advantage that it does not require only a sparse subset of all emitters to be active at any time, which promises significantly faster data acquisition rates.

**Figure 8:**
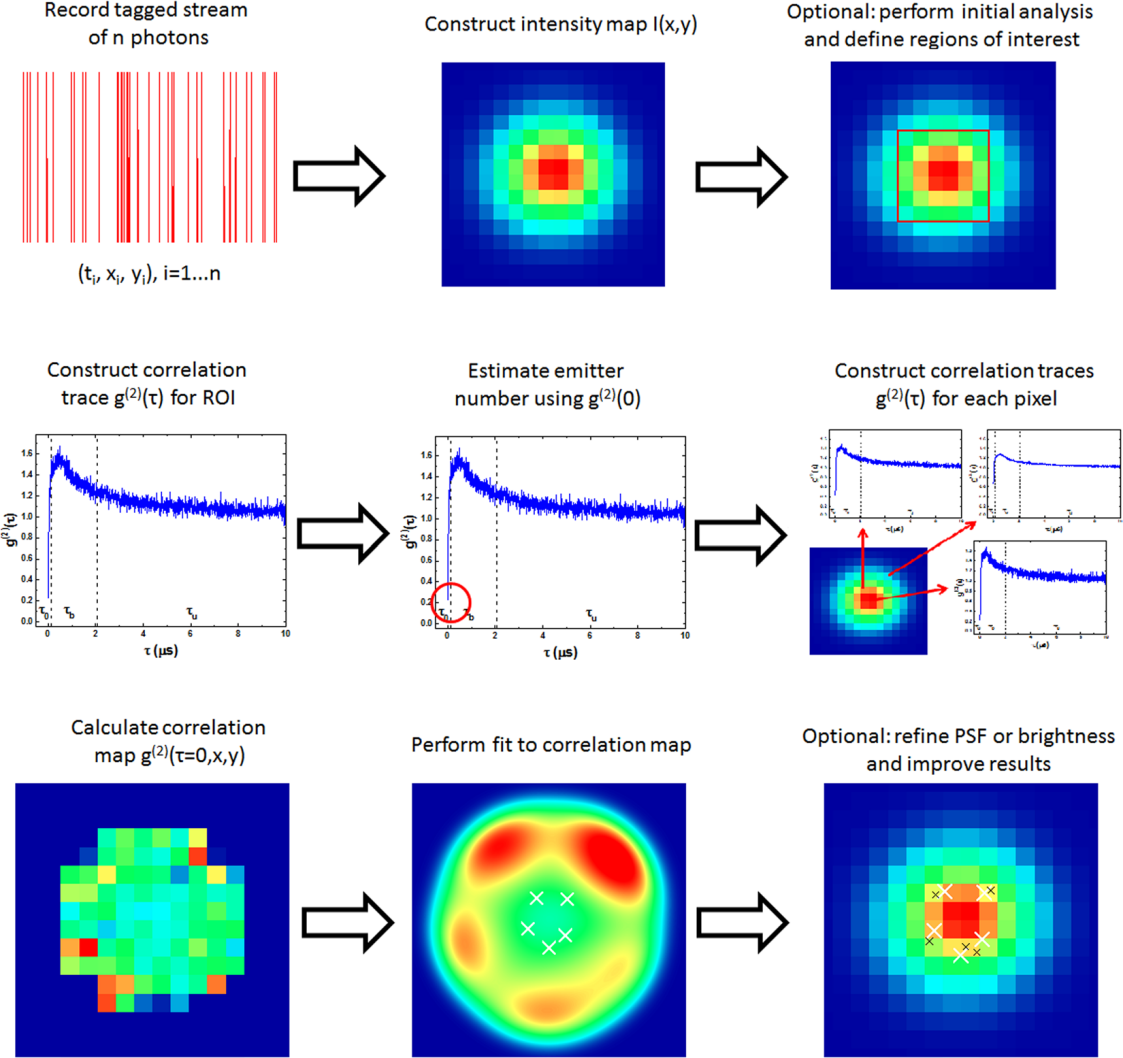
Schematic flowchart for QUEST including the steps where optimization or application of complementary technique seems appropriate.

In order to further understand the strengths and weaknesses of the present proposal and the differences compared to other super-resolution techniques, it is worthwhile to revisit the performance of QUEST in the presence of noise. As shown and discussed in Fig. 6, background noise may actually enhance the localization precision. The reason for this is that QUEST utilizes the normalized second-order correlation function, which is ideally completely independent of the intensity at any pixel. However in order to achieve good emitter number estimation and precise results at the fitting stage, information about the value of *g*^(2)^ at every pixel is required. This is a huge drawback for situations with strongly inhomogeneous illumination, such as having few emitters and regions in between with almost no signal intensity. In this case, a reasonable measurement of the value of *g*^(2)^ is achieved quite quickly at the emitter positions, but it takes very long for the pixels with low count rates to accumulate enough statistics to achieve low-error measurements of *g*^(2)^. As can be inferred from Fig. 6, a noise count rate between 500 and 1000 photons per pixel provides good results, which means that the signal peak count of 19242 photons is much more than what is required in order to achieve reasonable results. This also puts the quite large number of above 300000 photons needed to achieve good localization accuracy into perspective. For smaller regions or homogeneously illuminated detector pixels, the performance will improve significantly. These non-standard effects on the localization accuracy already show that the signal-to-noise ratio is not an ideal quantity in order to estimate how well QUEST performs, not only because of the non-standard interplay between noise and localization accuracy, but also because the temporal properties of the background noise distribution are more important than its mean value or variance. Considering a realistic implementation of the proposed technique, it will be necessary to find a suitable way to avoid the problem of regions with low intensity having a negative influence on localization accuracy. Neglecting pixels below some threshold intensity or using the mean intensity as a weight function are some basic possibilities, but their usefulness will depend strongly on the experimental conditions and are not optimal. It seems to be a more suitable approach to use QUEST in combination with another technique, using an algorithm which first applies the standard super-resolution technique and provides estimates of the spatially resolved emitter density and then applies QUEST selectively to these areas, in order to determine whether the high-density areas consist of several overlapping emitters. However, due to the huge number of possible combinations of techniques and ways to implement them, this is a problem best solved by machine learning or similar techniques. In order to visualize how QUEST works in general and to point out at which steps the addition of other techniques would be most beneficial, Fig. 8 shows a summarized schematic flowchart of the whole data analysis process. The steps, where optimization should take place are labeled as optional.

Still, the performance of QUEST is based on photon pairs and should therefore be inferior to other standard techniques in the limit of low count rates. Accordingly, at current it seems to be most promising to apply QUEST as an extension to other imaging techniques, which work well for isolated emitters, but have problems with distinguishing overlapping emitters at larger count rates. To this end, one would construct a superresolution image using a standard technique, use real-time statistical analysis on this image in order to identify regions, which are likely to have unidentified overlapping emitters and then apply QUEST to these regions. In order to be able to apply both the standard technique and QUEST to the same dataset and to achieve the temporal resolution necessary to identify the single emitter antibunching and estimate the number of emitters present, we suggest to use hardware based on the fiber-coupled single-photon avalanche detector array architecture, which has been introduced first for calorimetric purposes in particle physics^35^. Recently, the detector array technology has been improved significantly in terms of array size, readout rates and on-chip data processing^36,37^. In turn these technological advances have already led to first demonstrations of optimized super-resolution imaging by means of utilizing antibunching and postselection via a fiber bundle camera^13^. It offers low noise and negligible cross talk among pixels along with a large fill factor in conjunction with independently working detectors that can operate at a variable synchronized frame rate. Single photon avalanche diode arrays^12^ without fiber coupling are also a viable option, but typically suffer from rather large pixel sizes and small fill factors. We envision that by combining novel approaches in terms of detector hardware development, on-chip data processing and statistical analysis and optimized algorithms and by combining QUEST with other techniques, real-time super-resolution imaging may become feasible.

## Methods

### Simulation parameters

In order to model the emission of quantum dots, we adopted a standard power-law blinking model, where the on- and off-times are distributed with the probability *p*(*t*) = *t*^−*α*^ with exponents of *α*_*on*_ = 1.7 and *α*_*off*_ = 1.78. During on-times, waiting times for the next emitted photon are drawn randomly from an exponential distribution with a mean spontaneous emission time of 9 *μ*s. The position of detection for each photon on a grid with 1 nm resolution is modeled by a normalized symmetric two-dimensional Gaussian probability distribution with σ of 100 nm centered at the emitter which serves as the point spread function. To model the antibunching effect, we add another probability for the transition being blocked that depends on the time *τ* that has passed since the emission of the last photon: ${p}_{block}(t)=1-\exp (-\,\frac{\tau }{{\tau }_{block}})$ with a blockade time of *τ*_*black*_ = 30 ns. This total high-resolution image is then binned to pixels with a width of 50 nm each. To model the noise background, noise photons are added using an exponential distribution with a decay time of 5.5 *μ*s and assigned to a random pixel. For the noise series, we vary this decay time, but keep the total simulated duration constant.

### Data availability

The datasets generated during and/or analysed during the current study are available from the corresponding author on reasonable request.

## References

[1] Hell SW, Wichmann J (1994). Breaking the diffraction resolution limit by stimulated emission: stimulated-emission-depletion fluorescence microscopy. Opt. Lett..

[2] Klar TA, Hell SW (1999). Subdiffraction resolution in far-field fluorescence microscopy. Opt. Lett..

[3] Gustafsson MGL (2008). Three-dimensional resolution doubling in wide-field fluorescence microscopy by structured illumination. Biophys J.

[4] Betzig E (2006). Imaging intracellular fluorescent proteins at nanometer resolution. Science.

[5] Hess ST, Girirajan TPK, Mason MD (2006). Ultra-high resolution imaging by fluorescence photoactivation localization microscopy. Biophys J.

[6] Rust MJ, Bates M, Zhuang X (2006). Sub-diffraction-limit imaging by stochastic optical reconstruction microscopy (storm). Nat Meth.

[7] Lidke KA, Rieger B, Jovin TM, Heintzmann R (2005). Superresolution by localization of quantum dots using blinking statistics. Opt. Express.

[8] Holden SJ, Uphoff S, Kapanidis AN (2011). Daostorm: an algorithm for high- density super-resolution microscopy. Nat Meth.

[9] Huang F, Schwartz SL, Byars JM, Lidke KA (2011). Simultaneous multiple-emitter fitting for single molecule super-resolution imaging. Biomed. Opt. Express.

[10] Dertinger T, Colyer R, Iyer G, Weiss S, Enderlein J (2009). Fast, background-free, 3d super-resolution optical fluctuation imaging (sofi). Proceedings of the National Academy of Sciences.

[11] Glauber RJ (1963). The quantum theory of optical coherence. Phys. Rev..

[12] Vitali M (2014). A single-photon avalanche camera for fluorescence lifetime imaging microscopy and correlation spectroscopy. IEEE Journal of Selected Topics in Quantum Electronics.

[13] Israel Y, Tenne R, Oron D, Silberberg Y (2017). Quantum correlation enhanced super-resolution localization microscopy enabled by a fiber bundle camera. Nat. Commun..

[14] Kuno M, Fromm DP, Hamann HF, Gallagher A, Nesbitt DJ (2001). On/off fluorescence intermittency of single semiconductor quantum dots. The Journal of Chemical Physics.

[15] Efros AL, Nesbitt DJ (2016). Origin and control of blinking in quantum dots. Nat Nano.

[16] Basché T, Moerner WE, Orrit M, Talon H (1992). Photon antibunching in the fluorescence of a single dye molecule trapped in a solid. Phys. Rev. Lett..

[17] Lounis B, Moerner WE (2000). Single photons on demand from a single molecule at room temperature. Nature.

[18] Tinnefeld P, Müller C, Sauer M (2001). Time-varying photon probability distribution of individual molecules at room temperature. Chemical Physics Letters.

[19] Paul H (1982). Photon antibunching. Rev. Mod. Phys..

[20] Michler P (2000). A quantum dot single-photon turnstile device. Science.

[21] Weston KD (2002). Measuring the number of independent emitters in single-molecule fluorescence images and trajectories using coincident photons. Analytical Chemistry.

[22] Dong, C., Loy, C. C., He, K. & Tang, X. *Learning a Deep Convolutional Network for Image Super-Resolution*, 184–199 (Springer International Publishing, Cham, 2014).

[23] Zhu L, Zhang W, Elnatan D, Huang B (2012). Faster storm using compressed sensing. Nat Meth.

[24] Cox S (2012). Bayesian localization microscopy reveals nanoscale podosome dynamics. Nat Meth.

[25] Min J (2014). Falcon: fast and unbiased reconstruction of high-density super-resolution microscopy data. Sci Rep.

[26] Huang B, Wang W, Bates M, Zhuang X (2008). Three-dimensional super-resolution imaging by stochastic optical reconstruction microscopy. Science.

[27] Rozema LA (2014). Scalable spatial superresolution using entangled photons. Phys. Rev. Lett..

[28] Mahler B (2008). Towards non-blinking colloidal quantum dots. Nat Mater.

[29] Schwartz O, Oron D (2012). Improved resolution in fluorescence microscopy using quantum correlations. Phys. Rev. A.

[30] Schwartz O (2013). Superresolution microscopy with quantum emitters. Nano Letters.

[31] Geissbuehler S, Dellagiacoma C, Lasser T (2011). Comparison between sofi and storm. Biomed. Opt. Express.

[32] Cui J-M, Sun F-W, Chen X-D, Gong Z-J, Guo G-C (2013). Quantum statistical imaging of particles without restriction of the diffraction limit. Phys. Rev. Lett..

[33] Barsic A, Piestun R (2013). Super-resolution of dense nanoscale emitters beyond the diffraction limit using spatial and temporal information. Applied Physics Letters.

[34] Wang Y, Quan T, Zeng S, Huang Z-L (2012). Palmer: a method capable of parallel localization of multiple emitters for high-density localization microscopy. Opt. Express.

[35] Berglund S (2008). The atlas tile calorimeter digitizer. Journal of Instrumentation.

[36] Boiko DL (2009). A quantum imager for intensity correlated photons. New Journal of Physics.

[37] Burri S (2014). Architecture and applications of a high resolution gated spad image sensor. Opt. Express.

